# *IL18* gene variants rs187238 and rs360717 and their associations with psoriasis susceptibility and severity in a Brazilian case-control study^☆^

**DOI:** 10.1016/j.abd.2026.501364

**Published:** 2026-05-13

**Authors:** Cássio Rafael Moreira, Edna Maria Vissoci Reiche, Marcell Alysson Batisti Lozovoy, Andréa Name Colado Simão

**Affiliations:** a Department of Internal Medicine, Centro de Ciências da Saúde, Universidade Estadual de Londrina, Londrina, PR, Brazil; b Postgraduate Program in Clinical and Laboratory Physiopathology, Centro de Ciências da Saúde, Universidade Estadual de Londrina, Londrina, PR, Brazil; c School of Medicine, Universidade Católica do Paraná, Londrina, PR, Brazil; e Department of Pathology, Clinical Analysis and Toxicology, Centro de Ciências da Saúde, Universidade Estadual de Londrina, Londrina, PR, Brazil

Dear Editor,

Psoriasis (PsO) is a chronic inflammatory skin disease of immune-mediated origin, with an estimated prevalence between 1.0% and 3.0% in Western populations, and its pathophysiology involves a complex interaction between keratinocytes and components of the innate and adaptive immune systems.^1^ Several hypotheses have been formulated to elucidate the mechanisms underlying the pathogenesis of PsO; however, the prevailing theory proposes that an initial triggering event is followed by a sustained chronic inflammatory phase, regulated by a positive feedback loop, in which keratinocyte activation and proliferation is mediated by cytokines.^2^

Several studies have highlighted the relevance of Interleukin-18 (*IL-18*) in the pathophysiology of PsO. Patients with PsO have significantly elevated levels of *IL-18* in both skin lesions and serum, compared to healthy individuals.^3^ Previous investigations have demonstrated a correlation between plasma *IL-18* levels and disease severity, measured by the Psoriasis Area and Severity Index (PASI), suggesting that *IL-18* may act as a potential biomarker for PsO.^4^

Variations in genes encoding cytokines relevant to PsO may be associated with different biological processes and influence disease susceptibility. Single Nucleotide Variants (SNVs) in the *IL17A* and *IL17F*^5^ and *IL36G*^6^ genes have already been associated with predisposition to PsO. However, data on genetic variants of *IL18* in patients with PsO are scarce in the medical literature.

Thus, aiming to investigate the genetic variants of *IL18* and possible relationships with PsO susceptibility and severity, a case-control study was conducted that included, by convenience sampling, 256 individuals of both sexes, aged between 18 and 70 years, of which 128 patients were diagnosed with PsO recruited from the Dermatology Service of the Specialty Outpatient Clinic of Hospital Universitário (AEHU), Universidade Estadual de Londrina (UEL), Paraná, Brazil. The control group consisted of 128 healthy blood donors from the Regional Blood Center of Londrina, Paraná, Brazil.

Exclusion criteria included thyroid, renal, adrenal, hepatic, gastrointestinal, infectious, oncological diseases, as well as other autoimmune diseases. PsO severity was determined using the PASI at the initial diagnosis, prior to the start of topical and/or systemic treatments. The study was approved by the Research Ethics Committee on Human Beings of UEL under CAAE N. 37420820.0.0000.5231. All participants signed an Informed Consent Form.

Genomic DNA was extracted from the leukocyte layer of peripheral blood using a resin column procedure. Two SNVs of the *IL18* gene were genotyped: rs187238 C > G and IL18 rs360717 G > A. Genotyping of the variants was performed by quantitative real-time Polymerase Chain Reaction (qPCR), using the TaqMan™ method.

Hardy-Weinberg equilibrium was assessed and the frequencies and associations of *IL18* gene variants were analyzed according to allelic, dominant, codominant, recessive, and overdominant genetic models,^7^ using the online tool SNPStats (https://www.snpstats.net/start.htm).

To assess the effect of SNVs in the studied groups, binary logistic regression was performed, with the results expressed as Odds Ratio (OR) and 95% Confidence Interval (95% CI). The p-values ​​were adjusted for possible confounding variables, with statistical significance considered when p < 0.05.

The distribution of the genotypes of the *IL18* rs187238 C > G and *IL18* rs3607171 G > A variants was in Hardy-Weinberg equilibrium in both study groups (*IL18* rs187238 C > G: x^2^ = 0.7958, p = 0.3724 (control group) and x^2^ = 0.000, p = 1.000 (PsO group); *IL18* rs3607171 G > A: x^2^ = 0.0517, p = 0.8201 (control group) and x^2^ = 0.3178, p = 0.5729 (PsO group). As shown in Table 1, no statistically significant differences were observed in allelic or genotypic frequencies between patients with PsO and healthy controls in the different genetic models.

**Table 1:** Frequency distribution of *IL18* rs187238 C > G and *IL18* rs360717 G > A variant genotypes in healthy individuals and in individuals with psoriasis.

Model	Genotype	Control	Psoriasis	OR (95% CI)	p-value
**rs187238 C > G**					
Allelic	C	188 (73.4%)	192 (75%)	Reference	
	G	68 (26.6%)	64 (25%)	1.02 (0.61 ‒ 1.35)	0.837
Codominant	CC	71 (55.5%)	72 (56.3%)	Reference	
	CG	46 (35.9%)	48 (37.5%)	1.01 (0.59 – 1.72)	0.981
	GG	11 (8.6%)	8 (6.3%)	0.77 (0.29 – 2.06)	0.604
Dominant	CC	71 (55.5%)	72 (56.3%)	Reference	
	CG + GG	57 (44.5%)	56 (43.8%)	0.96 (0.57 – 1.58)	0.882
Recessive	GG	11 (8.6%)	8 (6.3%)	Reference	
	CG + CC	117 (91.4%)	120 (93.8%)	1.29 (0.49 – 3.44)	0.591
Overdominant	CC + GG	82 (64.1%)	80 (62.5%)	Reference	
	CG	46 (35.9%)	48 (37.5%)	1.04 (0.62 – 1.75)	0.886
**rs360717 (G > A)**					
Allelic	G	190 (74.2%)	191 (74.6%)	Reference	
	A	66 (25.8%)	65 (25.4%)	1.04 (0.65 ‒ 1.43)	0.879
Codominant	GG	71 (55.5%)	71 (55.5%)	Reference	
	GA	48 (37.5%)	47 (36.7%)	0.92 (0.54 – 1.57)	0.767
	AA	9 (7.0%)	10 (7.8%)	1.20 (0.45 – 3.17)	0.717
Dominant	GG	71 (55.5%)	71 (55.5%)	Reference	
	GA + AA	57 (44.5%)	57 (44.5%)	0.96 (0.57 – 1.58)	0.887
Recessive	AA	9 (7.0%)	10 (7.8%)	Reference	
	GA + GG	119 (93.0%)	118 (92.2%)	0.80 (0.31 – 2.08)	0.661
Overdominant	GG + AA	80 (62.5%)	81 (63.3%)	Reference	
	GA	48 (37.5%)	47 (36.7%)	0.90 (0.54 – 1.52)	0.699

*Note*: Data expressed as absolute numbers and percentages. Analyses adjusted for sex, age, and ethnicity.

OR, Odds Ratio; 95% CI, 95% Confidence Interval.

Additionally, the PsO group was divided according to disease severity, according to PASI (≤ 10 or > 10). No significant differences were identified between the different genotypes and alleles regarding this parameter (Table 2).

**Table 2:** Association between *IL18* rs187238 C > G and *IL18* rs360717 G > A variants and psoriasis activity, assessed by PASI ≤ 10 and PASI > 10.

Model	Genotype	PASI ≤ 10	PASI > 10	OR (95% CI)	p-value
rs187238 C > G					
Allelic	C	136 (73.1%)	56 (80%)	Reference	0.259
	G	50 (26.9%)	15 (20%)	0.68 (0.35 ‒ 1.33)	
Codominant	CC	50 (53.8%)	22 (62.9%)	Reference	
	CG	36 (38.7%)	12 (34.3%)	0.77 (0.33 – 1.79)	0.546
	GG	7 (7.5%)	1 (2.9%)	0.29 (0.33 – 2.52)	0.261
Dominant	CC	50 (53.8%)	22 (62.9%)	Reference	0.360
	CG + GG	43 (46.2%)	13 (37.1%)	0.68 (0.30 – 1.53)	
Recessive	GG	7 (7.5%)	1 (2.9%)	Reference	0.296
	CG + CC	86 (92.5%)	34 (97.1%)	3.10 (0.36 – 25.00)	
Overdominant	CC + GG	57 (61.3%)	23 (65.7%)	Reference	0.706
	CG	36 (38.7%)	12 (34.3%)	0.71 (0.85 – 1.96)	
rs360717 G > A					
Allelic	G	136 (73.1%)	53 (75.7%)	Reference	
	A	50 (26.9%)	17 (24.3%)	0.87 (0.46 ‒ 1.65)	0.674
Codominant	GG	51 (54.8%)	20 (57.1%)	Reference	
	GA	34 (36.6%)	13 (37.1%)	1.05 (0.45 ‒ 2.43)	0.913
	AA	8 (8.6%)	2 (5.7%)	0.57 (0.11 – 3.00)	0.511
Dominant	GG	51 (54.8%)	20 (57.1%)	Reference	
	GA + AA	42 (45.2%)	15 (42.9%)	0.94 (0.42 – 2.12)	0.892
Recessive	AA	8 (8.6%)	2 (5.7%)	Reference	
	GA + GG	85 (91.4%)	33 (94.3%)	1.78 (0.35 – 9.09)	0.489
Overdominant	GG + AA	59 (63.4%)	22 (62.9%)	Reference	
	GA	34 (36.6%)	13 (37.1%)	1.12 (0.49 – 2.55)	0.793

Data expressed as absolute numbers and percentages. Analyses adjusted for sex, age, and ethnicity.

OR, Odds Ratio; 95% CI, 95% Confidence Interval.

IL-18 has been shown to favor the differentiation and maintenance of T-helper (Th)-17 cells and that the use of neutralizing antibody against IL-18 was able to inhibit the Th17 immune response in a murine model of PsO.^8^ These findings indicate that the IL-18-mediated immune response may play a relevant role in PsO pathogenesis, and its inhibition is considered a potential therapeutic strategy.

The present study found no significant differences between *IL18* gene variants and PsO susceptibility or severity. Conversely, a study conducted in the Japanese population identified a significantly higher frequency of the G genotype of the rs187238 variant in individuals with PsO, suggesting that this genotype may be associated with greater functional activity in inducing IL-18 production.^9^ From what is known in the medical literature to date, no studies have been described that explore the association between the *IL18* variant rs360717 and PsO.

Similar investigations have been conducted in other dermatological diseases. A meta-analysis with quantitative data suggested that the genetic variant rs187238 of the *IL18* gene may influence the risk of developing atopic dermatitis in the general population.^10^

In conclusion, this is the first Brazilian study to investigate the possible association between the variants rs187238 and rs360717 of the *IL18* gene and PsO. Although the results did not demonstrate a significant association between these genetic variants and disease susceptibility or severity, the data contribute in an unprecedented way to the understanding of the genetic basis of PsO in the Brazilian population. These findings reinforce the importance of further studies to better clarify the role of IL-18 in the pathophysiology of PsO and its potential as a biomarker.

## ORCID IDs

Cássio Rafael Moreira: 0000-0002-8781-1505

Edna Maria Vissoci Reiche: 0000-0001-6507-2839

Marcell Alysson Batisti Lozovoy: 0000-0002-4023-9548

Andréa Name Colado Simão: 0000-0002-2073-6782

## Research data availability

The entire dataset supporting the results of this study was published in this article.

## Financial support

This study was funded by the Coordination for the Improvement of Higher Education Personnel (CAPES), Ministry of Education, Brazil, and by the Institutional Program for Undergraduate Research Scholarships (PIBIC) of the National Council for Scientific and Technological Development (CNPq), Brazil.

## Authors' contributions

Cássio Rafael Moreira: Design and planning of the study; collection; analysis and interpretation of data; drafting and editing of the manuscript; critical review of the manuscript; approval of the final version of the manuscript.

Edna Maria Vissoci Reiche: Design and planning of the study; statistical analysis; analysis and interpretation of data; drafting and editing of the manuscript; critical review of the manuscript; approval of the final version of the manuscript.

Marcell Alysson Batisti Lozovoy: Design and planning of the study; laboratory analysis; statistical analysis; analysis and interpretation of data; drafting and editing of the manuscript; critical review of the manuscript; approval of the final version of the manuscript.

Andréa Name Colado Simão: Design and planning of the study; laboratory analysis; statistical analysis; analysis and interpretation of data; drafting and editing of the manuscript; critical review of the manuscript; approval of the final version of the manuscript.

## Conflicts of interest

None declared.
